# Supplementary material for: A correlation map of genome-wide DNA methylation patterns between paired human brain and buccal samples
Source: Clin Epigenetics. 2022 Nov 1;14:139. doi: 10.1186/s13148-022-01357-w (PMC9628033; doi:10.1186/s13148-022-01357-w)
Supplement: Supplementary file 2 — Additional file 2. Supplementary Figures. [file 13148_2022_1357_MOESM2_ESM.docx]

# Supplementary Figures


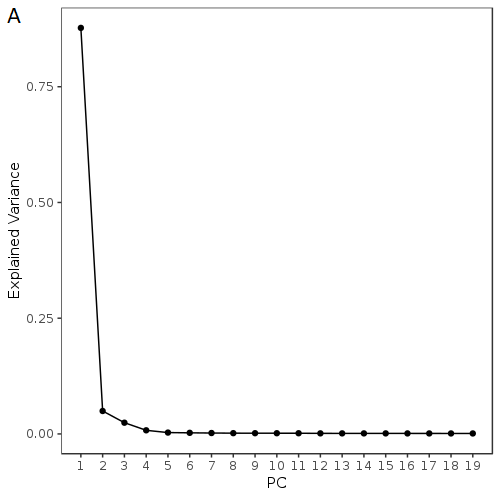

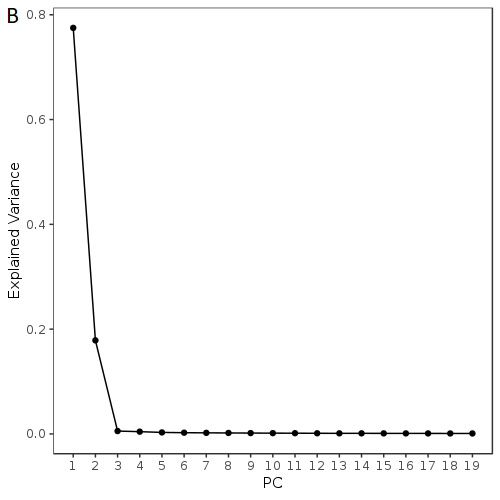

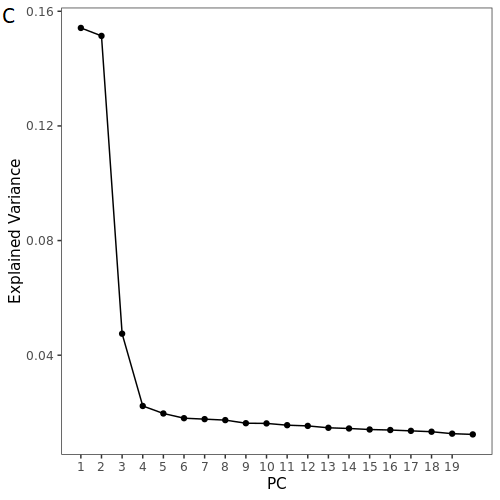

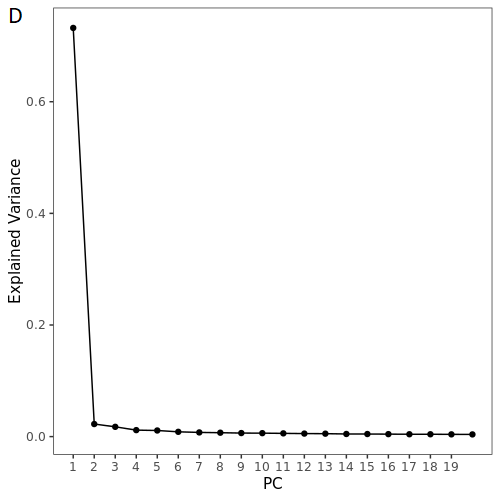


*S1: Scree plots of PCA of normalized DNAm beta values in MADRC-1 brain (A, DNAm PC1 was used for analyses), buccal (B, DNAm PC 1 and 2 were used for analyses), MADRC-2 brain (C, DNAm PC1 to 3 were used for analyses), buccal (D, DNAm PC 1 was used for analyses)*

*
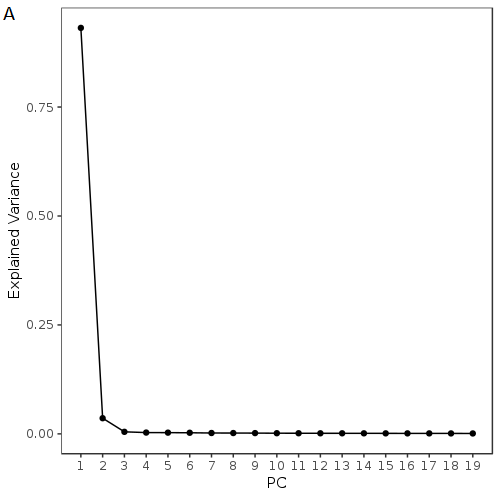

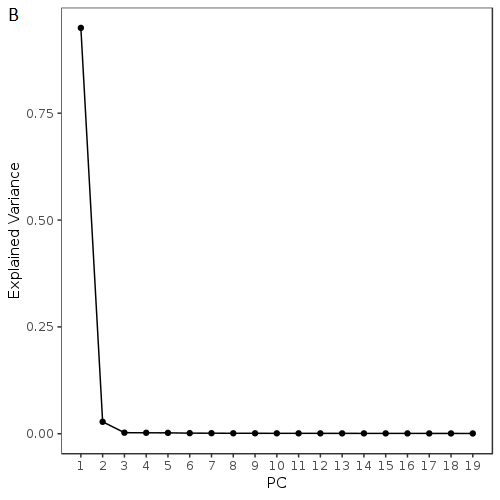
*

*S2: Scree plots of PCA of normalized DNAm beta values in the Braun et al. data for brain (A, DNAm PC 1 was used for analyses) and buccal (B, DNAm PC 1 was used for analyses) samples*


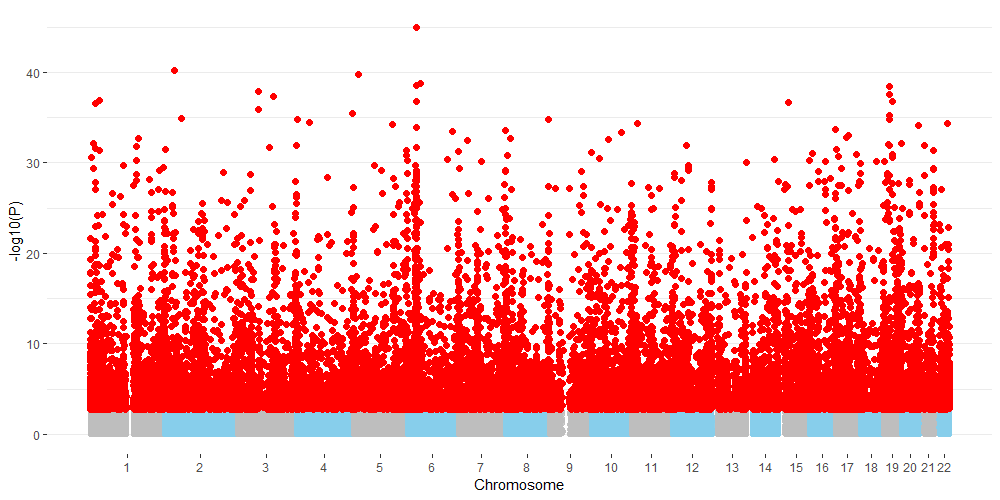


*S3: Manhattan plot of the Spearman rank correlation test results. P-values are plotted and significantly correlated CpG sites after FDR multiple testing correction (q < 0.05) are highlighted in red.*


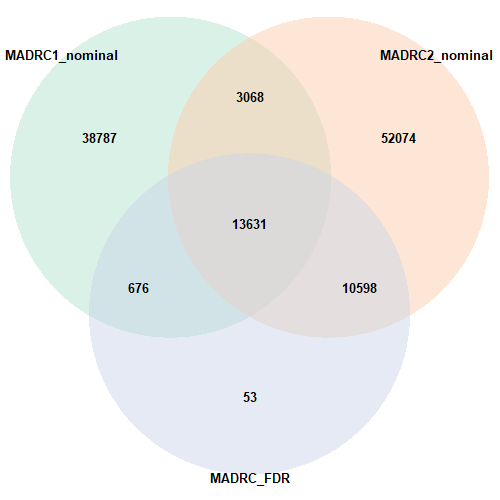


*S4: Venn diagram displaying the overlap between CpG probes that are correlated between PFC and buccal samples with a nominal p-value below 0.05 in the analyses in each individual batch (green and red), and the significantly correlated CpG sites in the combined analysis (purple).*


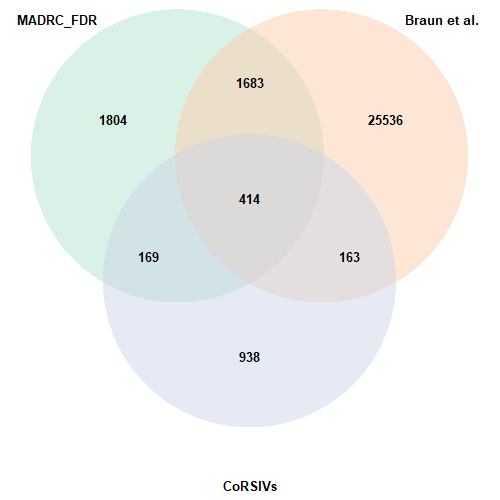


*S5: Venn diagram showing significantly correlated CpG sites between PFC and buccal samples after multiple testing correction in the MADRC dataset and filtering for correlation coefficients R < -0.5 or R>0.5 (resulting in n=4,070 correlated CpGs). Overlap of significantly correlated CpG sites between PFC and buccal samples (FDR q < 0.05; green) with mQTLs according to the DLPFC mQTL database (FDR q < 0.05; orange)* (33) *and the buccal mQTL database generated from an independent dataset ([unpublished data], FDR q < 0.05; violet).*


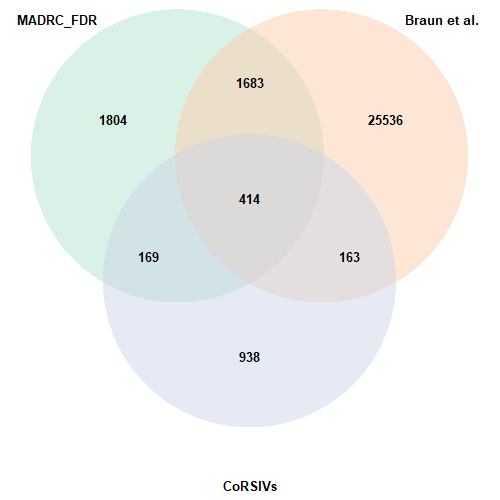


*S6: Venn diagram comparing significantly correlated CpG sites with a Spearman rank correlation coefficient R < -0.5 or R>0.5 between buccal and brain samples in our analysis (MADRC; green), the analysis using Braun et al.* (17) *data (orange), and CoRSIVs (violet)* (19)*.*


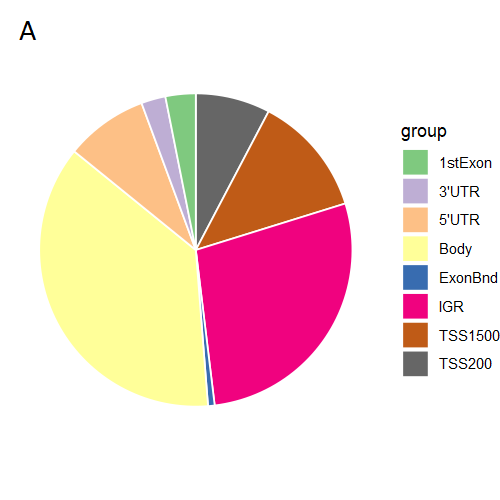

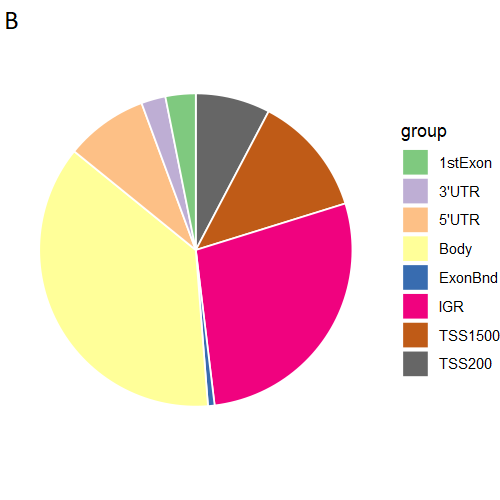

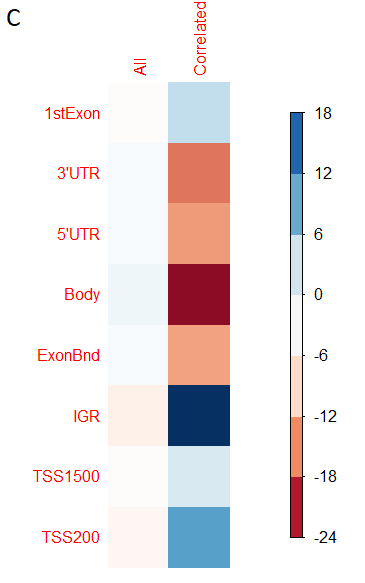


*S7: Enrichment of genomic regions using a Spearman rank correlation coefficient R< -0.5 or R>0.5 between buccal and brain samples in the MADRC dataset; Panel A: Distribution of genomic regions in all tested CpG sites on the EPIC array; B: Distribution of genomic regions in significantly correlated (FDR q < 0.05) CpG sites; Panel C: Pearson’s residuals of the Chi-squared test, blue indicates an enrichment and red a depletion of the respective genomic region*
